# Roles of C-Terminal Region of Yeast and Human Rad52 in Rad51-Nucleoprotein Filament Formation and ssDNA Annealing

**DOI:** 10.1371/journal.pone.0158436

**Published:** 2016-06-30

**Authors:** Nilesh V. Khade, Tomohiko Sugiyama

**Affiliations:** Department of Biological Sciences, Ohio University, Athens, Ohio, United States of America; University of California, San Francisco, UNITED STATES

## Abstract

Yeast Rad52 (yRad52) has two important functions at homologous DNA recombination (HR); annealing complementary single-strand DNA (ssDNA) molecules and recruiting Rad51 recombinase onto ssDNA (recombination mediator activity). Its human homolog (hRAD52) has a lesser role in HR, and apparently lacks mediator activity. Here we show that yRad52 can load human Rad51 (hRAD51) onto ssDNA complexed with yeast RPA *in vitro*. This is biochemically equivalent to mediator activity because it depends on the C-terminal Rad51-binding region of yRad52 and on functional Rad52-RPA interaction. It has been reported that the N-terminal two thirds of both yRad52 and hRAD52 is essential for binding to and annealing ssDNA. Although a second DNA binding region has been found in the C-terminal region of yRad52, its role in ssDNA annealing is not clear. In this paper, we also show that the C-terminal region of yRad52, but not of hRAD52, is involved in ssDNA annealing. This suggests that the second DNA binding site is required for the efficient ssDNA annealing by yRad52. We propose an updated model of Rad52-mediated ssDNA annealing.

## Introduction

Faithful repair of DNA double-strand breaks (DSBs) is crucial for genome stability. An unrepaired DSB may cause cell death and erroneous DSB repair may cause cancer [1–3]. Rad52 is one of the key proteins involved in DSB repair [4, 5]. The role of Rad52 in the DSB repair has been extensively studied in the yeast *Saccharomyces cerevisiae*, where Rad52 is required for DSB repair both in the high-fidelity homologous recombination (HR) pathway [6–9] and in an error-prone single strand annealing (SSA) pathway [10]. *In vitro* studies have shown that Rad52 can associate with single-stranded DNA (ssDNA) that is complexed with the ssDNA binding protein RPA [11, 12], and mediates two reactions that are crucial for HR and SSA pathways. In the HR pathway, Rad52 recruits Rad51, a RecA-family recombinase onto ssDNA [13, 14]. The resulting Rad51-ssDNA nucleoprotein filament catalyzes DNA strand invasion, a key step of HR pathway [15]. The recruitment of Rad51 onto ssDNA is termed recombination mediator activity [4]. In addition, Rad52 can anneal complementary ssDNA molecules in the SSA pathway [16].

Rad52 is conserved from yeast to human, with homology mainly in the N-terminal two thirds of the protein [17]. The conserved region is essential for the function of Rad52, and referred to as Rad52NM [18]. For clarity, after this point, yeast proteins are denoted with prefix “y” (e.g. yRad52), and human proteins are denoted with prefix “h” and capitalized (e.g. hRAD52). Both yRad52NM and hRAD52NM are involved in binding ssDNA, RPA, and second molecule of Rad52 [18–23]. Consistently, both yRad52NM and hRAD52NM can mediate ssDNA annealing. However, yRad52 cannot anneal ssDNA that is complexed with hRPA, indicating that yRad52-yRPA interaction is species-specific [11, 24].

The role of the C-terminal region of Rad52 seems to be considerably different in yeast and mammals. The C-terminal region (amino acids 327–504) of yRad52 is involved in protein-protein interaction with yRad51, and is required for mediator activity [18, 19, 25, 26]. In contrast, attempts to detect recombination mediator activity of hRAD52 *in vitro* have been unsuccessful [4, 27], although hRAD52 has the ability to bind hRAD51 *in vitro*. Consistent with the *in vitro* observations, the impact of the loss of *RAD52* function in mammalian cells is less pronounced than in yeast. *RAD52*^-/-^ mouse ES cells showed only moderate reduction in homology-dependent gene-targeting. *RAD52*^-/-^ ES cells were not hypersensitive to X-ray or MMS. Furthermore, *RAD52*-knockout mice were viable, fertile, and had normal immune systems [28].

BRCA2, not Rad52, seems to be a major mediator protein in mammalian cells [29, 30]. hBRCA2 binds hRAD51 through a short peptide (approximately 30 amino acids) repeat sequence, the BRC repeat [31]. Interestingly, the BRC repeat does not have any clear sequence similarity to the hRAD51-binding region of hRAD52. This suggests that hRAD52 and hBRCA2 have distinct hRAD51 binding mechanisms. In this study, we demonstrate that the mediator activity of yRad52 recruits hRAD51 onto ssDNA *in vitro*. Then we analyzed the roles of the C-terminal regions of yRad52 and hRAD52 in mediator activity and ssDNA annealing. We also constructed Rad52-BRC repeat fusion proteins and explored whether the BRC repeat can substitute the C-terminal region of Rad52 and thereby stimulate the DNA strand exchange by hRAD51.

## Materials and Methods

### Plasmids

The plasmids for overexpression of hRAD51 (phRad51.1), hRPA (p11d-tRPA), and yRad52 (pQE60-yRad52) were obtained from Patrick Sung (Yale University), Mark Wold (University of Iowa), and Rodney Rothstein (Columbia University), respectively. Sequences of synthetic DNA that were used in this study are shown in S1 Table. All Rad52 variants were expressed with a C-terminal hexa-histidine (His_6_) tag. pQE60-yRad52NM was constructed from pQE60-yRad52 by deleting a *Bam*HI fragment that encoding C-terminal region *RAD52* ORF. pET21-yRad52NM-BRC4 was constructed as follows. First, a double-strand DNA (dsDNA) encoding the BRC4 polypeptide (35 amino acids) was produced by hybridization of two synthetic DNA molecules (Pri-1 and 2) followed by PCR amplification. The dsDNA product was inserted at the *Bam*HI site of pQE60-yRad52NM. The resulting plasmid was used as a template for PCR (with Pri-3 and 4) to amplify the yRad52NM-BRC4 ORF, and the PCR product was inserted between the *Nde*I and *Xho*I sites of pET21a. pET21-yRad52NM-BRC3-4 was constructed as follows. First, to create an *Xho*I site just after the *Bam*HI site of yRad52NM encoding region, pQE60-yRad52NM was amplified by PCR (with Pri-5 and 6) and cloned between *Nde*I and *Xho*I sites of pET21a. The resulting plasmid was digested with *Bam*HI and *Xho*I and ligated with two synthetic dsDNAs (BRC3 and BRC4a) that encode BRC3 (35 amino acids) and BRC4. pET21-yNM-BRC4_X3_ containing three BRC4 repeats was constructed by ligating three synthetic dsDNA (BRC4b) encoding BRC4 into the *Bam*HI site of pQE60-yRad52NM, and the fused ORF was amplified by PCR (with Pri-7 and 8) and cloned between *Nde*I and *Xho*I sites of pET21a. Human *RAD52* cDNA was obtained from PlasmID (Harvard Medical School) and amplified by PCR (with Pri-9 and 10) and cloned between *Nde*I and *Xho*I sites in pET21a to create pET21-hRAD52. Sequence alignment indicated that the region from the 1st to the 286th amino acid of hRAD52 corresponded to yRad52NM (1-327th; S1 Fig). To create pET21-hRAD52NM, a DNA segment encoding 1–290 amino acids (hRAD52NM) was amplified (with Pri-9 and 11) and cloned between the *Nde*I and *Xho*I sites in pET21a. pET21-hRAD52NM-BRC4 was constructed by inserting the synthetic dsDNA (BRC4b) encoding the BRC4 peptide into the *Bam*HI site in pET21-hRAD52NM. All newly constructed plasmids were confirmed by sequencing.

### Purification of proteins

All Rad52 derivatives were expressed as C-terminal His_6_-tag fusion proteins. yRad52 was purified as previously published [16] with modifications as described [12]. yRad52NM, yRad52NM-BRC4 and yRad52NM-BRC3-4 were overexpressed by IPTG in *E*. *coli* BL21(DE3) harboring pQE60-yRad52NM, pET21-yRad52-BRC4, and pET21-yRad52-BRC3-4, respectively, and purified by the same protocol [12]. hRAD52, hRAD52NM and hRAD52NM-BRC4 were overexpressed by IPTG in BL21(DE3) harboring pET21-hRAD52, pET21-hRAD52NM, and pET21-hRAD52NM-BRC4, respectively, and purified as described [12] except that Heparin column chromatography was omitted.

yRad52NM-BRC4_X3_ was overexpressed by IPTG in BL21(DE3) harboring pET21-yRad52NM-BRC4_x3_ and purified as follows. First, cells were suspended in lysis buffer (30 mM Tris-HCl (pH 7.5), 5% (v/v) glycerol and 500 mM NaCl) containing 10mM imidazole, 1 mM PMSF, and a protease inhibitor cocktail (Roche) and lysed using a Branson Sonifier 250. Insoluble cell debris was removed by centrifugation at 20,000 rpm for 30 min with a Beckman JA25.50 rotor. The cleared lysate was loaded on a nickel (Ni) Sepharose column (7 ml; GE Healthcare). The yRad52NM-BRC4_X3_ protein did not bind to the resin. Thus the flow through (FT) was diluted with an equal volume of TDEG buffer (30 mM Tris-HCl (pH7.5), 1 mM dithiothreitol, 1 mM EDTA, 5% (v/v) glycerol) containing no NaCl and loaded onto a Heparin Sepharose column (5 ml; GE Healthcare) that was pre-equilibrated with TDEG buffer containing 100 mM NaCl. The column was washed with TDEG buffer containing 100 mM NaCl, and then subjected to a linear gradient from 100 mM to 1M NaCl in TDEG buffer. The fractions containing yRad52NM-BRC4_X3_ (eluted with approximately 800 mM NaCl) were pooled and dialyzed for 3 hours (2x) against TGEB buffer (30 mM Tris-HCl (pH7.5), 5 mM 2-mercaptoethanol, 1 mM EDTA, 5% (v/v) glycerol) containing 100 mM NaCl, and loaded on the Mono-Q column (1ml; GE Healthcare) equilibrated with the TGEB buffer containing 100mM NaCl. The column was washed with the same buffer and subjected to a linear gradient from 100 mM to 1 M NaCl in the TGEB buffer. The fractions containing yRad52NM-BRC4_X3_ were pooled and loaded onto a 1 ml Ni-Sepharose column that was equilibrated with 50 mM Tris-Cl (pH7.5), 500 mM NaCl, 5% (v/v) glycerol, and 10 mM imidazole (I-10 buffer). The column was washed with I-10 buffer and subjected to a step gradient of 50 mM and 200 mM imidazole in I-10 buffer. The yRad52NM-BRC4_X3_ was eluted with 200 mM imidazole and concentrated with an Amicon ultra 30 (Millipore) to 20.4 μM, and stored at -80°C.

yRad51 was purified as described [32]. hRAD51 was expressed in *E*. *coli* BLR(DE3) (pLysS) harboring phRAD51.1 as described [33], and purified as follows. Harvested cells (60 g) were suspended in 180 ml of TGEB containing 100 mM NaCl, 1 mM PMSF, and a protease inhibitor cocktail (Roche) and lysed using a Branson Sonifier 250. The cell debris was cleared by centrifugation at 20,000 rpm for 30 min using a Beckman JA25.50 rotor. The cleared lysate was then loaded onto a Q-Sepharose FF column (30 ml; GE Healthcare) that was pre-equilibrated with TGEB buffer containing 100 mM NaCl. The column was washed with TGEB buffer containing 100 mM NaCl and the protein was eluted using a linear gradient of 100 mM to 1 M NaCl in 150 ml TGEB buffer. The fractions containing hRAD51 were pooled and dialyzed against MDG-200 buffer (20 mM K-4-morpholineethanesulfonic acid (MES; pH 6.5), 1 mM dithiothreitol, 5% (v/v) glycerol, 10 mM potassium phosphate, and 200 mM NaCl) for 3 hrs (2x), and loaded onto a hydroxyapatite (HAP) column (9 ml; BioRad) equilibrated with MDG-200 buffer. hRAD51 was found in the flow through, which was then loaded onto a Heparin column (5 ml; GE Healthcare) equilibrated with TDEG buffer containing 100 mM NaCl. The column was washed with the same buffer and then subjected to a linear gradient from 100 mM to 1 M NaCl in TDEG buffer. The fractions containing the hRAD51 were pooled, dialyzed against MDG-200 buffer, and then loaded onto a HAP column (9 ml; BioRad) equilibrated with MDG-200 buffer. The hRAD51 bound to HAP under these conditions. The column was washed with the same buffer and subjected to a linear gradient from 10 mM to 400 mM potassium phosphate in MDG-200 buffer. The fractions containing the hRAD51 were pooled and diluted with equal amount of TGEB buffer and loaded onto a Mono-Q column (1 ml; GE Healthcare) equilibrated with TGEB buffer containing 100 mM NaCl. The column was washed with the same buffer and subjected to a linear gradient from 100 mM to 1 M NaCl. The fractions containing the hRAD51 were pooled and concentrated to 21.2 μM with an Amicon ultra 30, divided into small aliquots, and stored at -80°C.

The yeast strain to overexpress yRPA was obtained from Richard Kolodner (University of California, San Diego) and yRPA was purified as described [34]. hRPA was overexpressed in BLR(DE3)(pLysS) harboring p11d-tRPA as described [35]. Cells were suspended in the cell disruption buffer (20 mM Tris-HCl (pH7.5), 20 mM EDTA, 5 mM 2-mercaptoethanol, 5% (v/v) glycerol, and 0.5 M NaCl) containing 1 mM PMSF, lysed with glass beads, and cleared as described above. The lysate was loaded onto a ssDNA cellulose column (30 ml) that was equilibrated with buffer-A (25 mM Tris-HCl (pH7.5), 5 mM EDTA, 5 mM 2-mercaptoethanol, and 5% (v/v) glycerol) containing 0.5 M NaCl. The column was washed with 100 ml of buffer-A containing 0.75 M NaCl. The hRPA was eluted with buffer-A containing 1.5 M NaCl and 50% (v/v) ethylene glycol. The hRPA (40 ml) was then de-salted by passage through a 200 ml G-25 column (GE Healthcare) that was equilibrated with MDG-200 buffer containing 5 mM K-phosphate, and then loaded on a HAP column (10 ml) that was equilibrated with the same buffer. The column was washed with MDG-200 buffer containing 5 mM K-phosphate and then subjected to a linear gradient from 5 to 100 mM K-phosphate in MDG-200 buffer. The fractions containing hRPA (eluted with approximately 80 mM phosphate), were pooled and mixed with a 2x volume of buffer-A, and then loaded onto a mono-Q column (1 ml; GE Healthcare) equilibrated with buffer-A containing 50 mM NaCl. The column was washed with buffer-A containing 50 mM NaCl, and then subjected to a linear gradient from 50 to 500 mM NaCl in buffer-A. The hRPA eluted from the column with approximately 240 mM NaCl. The protein was dialyzed against buffer-A containing 50 mM NaCl, concentrated to 6.77 μM using Amicon-ultra 30, divided in small aliquots and stored at -80°C. Protein concentrations were determined using a Coomassie protein assay kit (Pierce).

### DNA binding Assay

The 70-nt ssDNA (TSO252, S1 Table) was labeled with ^32^P using T4 polynucleotide kinase (NEB) and γ-^32^P-ATP (Perkin Elmer). Rad52 or its derivatives was incubated with 100 nM of the labeled TSO252 at 37°C for 15 minutes in a 10 μl reaction mixture containing 30 mM TrisAc (pH7.5), 3 mM MgAc, and 20 mM NaCl. The sample was then mixed with 4 μl of TAE loading dye (50% (v/v) glycerol and 0.1% bromophenol blue in Tris-acetate EDTA buffer), loaded onto a 6% polyacrylamide gel (19 cm x 16 cm) in Tris-borate EDTA buffer, and separated by electrophoresis at 200 V. The gel was dried on DE81 chromatography paper (Whatman) and the labeled products were visualized using a BioRad Personal FX phosphor imager. Radioactive signals were quantified using Quantity One software (BioRad), and the percentage of the Rad52-ssDNA complex in reference to total radioactivity in each lane was determined.

### Mediator assay

ΦX174 phage circular ssDNA and dsDNA were purchased from New England Biolabs and the dsDNA was linearized using *Xho*I. RPA and ssDNA were incubated for 2 minutes at 37°C in buffer containing 40 mM Tris-HCl (pH 7.5), 1 mM DTT, 2 mM ATP, 1 mM MgCl_2_, 8 mM creatine phosphate, and 28 μg/ml creatine phosphokinase. Then Rad52 or its derivatives was added to the reaction and incubation continued for 3 min. Then hRAD51 was added to start hRAD51-ssDNA filament formation. After 5 minutes, linear dsDNA, ammonium sulfate (to 100 mM), and spermidine (to 4 mM) were added to start DNA strand exchange. The volume of the reaction at this point was 15 μl, in which the final concentrations of RPA, hRAD51, ssDNA, and dsDNA were 2.65 μM, 7.5 μM, 15 μM (nt), and 15 μM (bp), respectively. After 90 minutes at 37°C, the reaction was stopped and deproteinized by adding 5 μl of stop buffer (10% SDS, 10 mg/ml proteinase K in TAE dye) and incubating for another 15 minutes. The DNA products were separated by 1% agarose gel electrophoresis (20 x 26 cm) in TAE buffer at 35 V for 15 hours. The gel was stained with ethidium bromide (1 μg/ml) in TAE for 1 hour and destained in H_2_O for another 1 hour. DNA bands were visualized with BioRad ChemiDoc XRS+ imaging system and quantified using Image Lab software. DNA strand exchange was quantified as percentage of products (sum of the nicked circle dsDNA and the joint molecules) in total DNA each lane. Then, relative DNA strand exchange was expressed as a value relative to the control reactions carried out in the absence of the potential mediator. For better band visualization, black and white images of the gels were inverted in the figures.

### Annealing assay

A fluorescence-based annealing assay was performed in a 400 μl reaction essentially as described [24]. First, two complementary 70-mer ssDNA oligos TSO252 and TSO253 (40 μM (nt)) were incubated with or without RPA (36 nM) in a standard reaction mixture (30 mM TrisAc (pH 7.5), 5 mM MgAc, and 0.4 μM 4',6-diamidino-2-phenylindole (DAPI)). The annealing reaction was started by adding Rad52 derivatives. DAPI fluorescence was monitored every second by using a HORIBA Jobin Yvon FluoroMAX-3 spectrofluorometer. The annealing rates were determined from the linear portions of the slopes of fluorescence/time curves (between 0.33 and 2 minutes).

### Protein-protein binding assay

hRAD51 (100 pmoles) and His_6_-tagged yRad52 (40 pmoles) were incubated for 10 min at 37°C in 30 μl of buffer containing 30 mM Tris-acetate (pH7.5), 5 mM Mg-acetate, 0.03% Igepal, 2.5 mM ATP, and 10 μl (bed volume) of Ni-Sepharose beads. Reaction mixture was then centrifuged at 800 xg for 3 min, and beads fraction was washed with 400 μl of 30 mM Tris-acetate (pH 7.5), 10 mM imidazole, 50 mM NaCl, 5 mM Mg-acetate, 0,03% Igepal, and 1 mM ATP three times. Bound proteins were eluted in 20 μl of 1x SDS loading dye and analyzed by 10% SDS-PAGE. Gel was stained with Coomassie brilliant blue R-250.

## Results

### Yeast Rad52 recruits human Rad51 onto ssDNA that is complexed with yeast RPA *in vitro*

**Fig 1:**
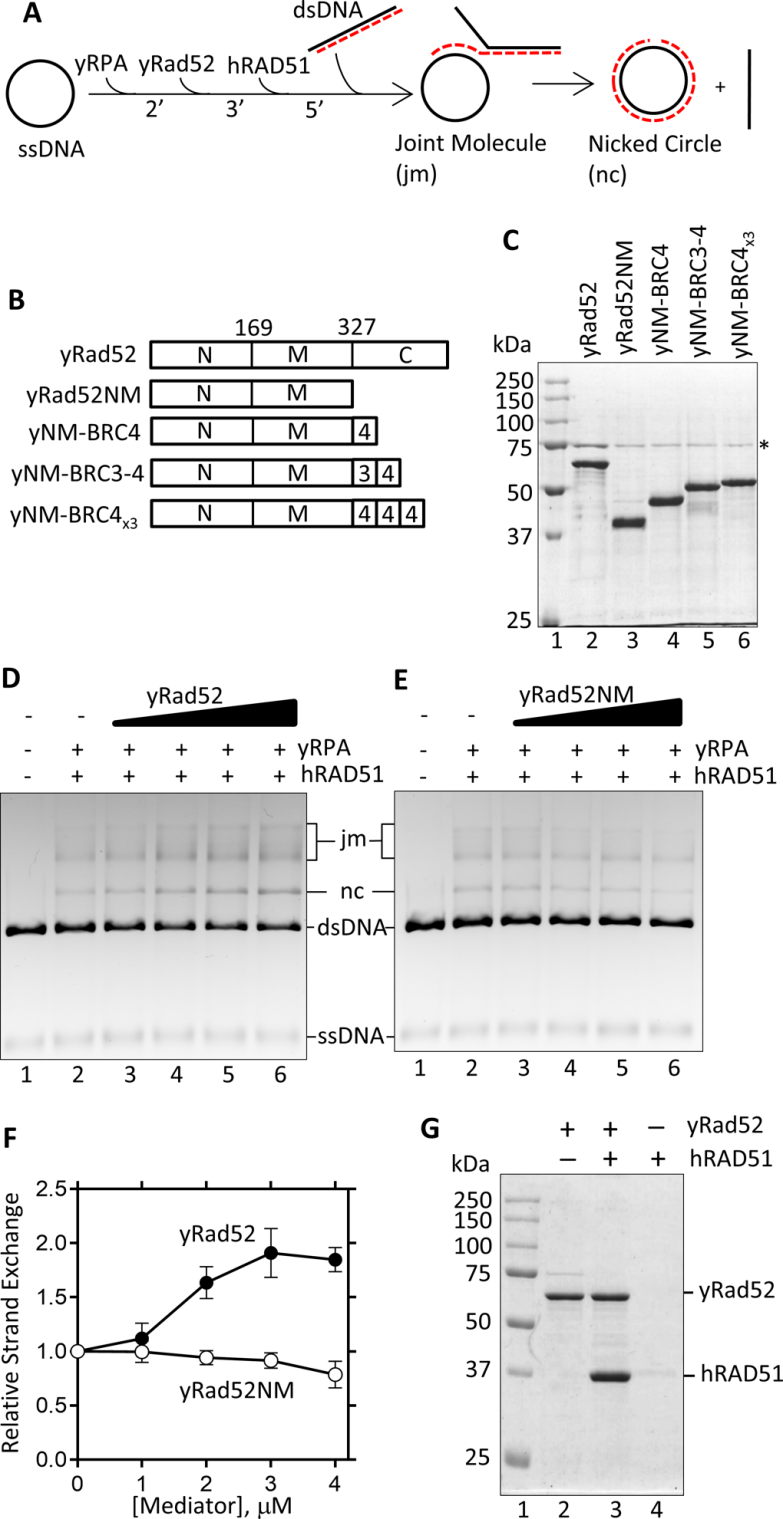
Mediator assay of yRad52 with hRAD51. (A) Illustration of the DNA strand exchange experiment to analyze the mediator activity. ΦX-174 ssDNA was incubated with yRPA, yRad52, hRAD51, and then with ΦX-174 dsDNA in the indicated order. The reaction produced joint molecules (jm) and a nicked circular (nc) dsDNA. (B) Derivatives of yRad52. N-terminal (N), middle (M), and C-terminal (C) region have been described in previous paper [18]. For bottom three constructs, yRad52NM was fused with a human BRC4 (NM-BRC4), BRC3-BRC4 (NM-BRC3-4), or three repeats of BRC4 (NM-BRC4_x3_). (C) Same amount (2 μg) of purified yRad52 and its derivatives were separated by SDS-PAGE and stained with coomassie brilliant blue. Asterisk indicates a contaminating protein present in all preparations. (D and E) DNA strand exchange was performed in the absence (lane 2) or presence of 1, 2, 3, 4 μM (lane 3 to 6) of yRad52 (D) or yRad52NM (E). DNA products were separated through agarose gel and visualized with ethidium bromide staining. Lane 1 shows a control reaction without any protein. (F) The products (nicked circles (nc) and joint molecules (jm)) were quantified from D (yRad52) and E (yRad52NM) and repeated experiments and relative product formation was plotted against the mediator concentration. Product formation in the absence of the mediator was 31.2%, which was defined as 1.0. Error bars are standard deviations (n = 3). (G) hRAD51 and His-tagged yRad52 were mixed as indicated and precipitated with Ni-beads. Proteins on the beads were then eluted and analyzed by SDS-PAGE.

Although yRad52 and hRAD52 share 34% amino acid similarity, attempts to detect recruitment of hRAD51 onto hRPA-ssDNA complex by hRAD52 (mediator activity) have been unsuccessful [4, 27]. To understand the apparent lack of hRAD52 mediator activity, we first investigated whether yRad52 can recruit hRAD51 onto a yRPA-ssDNA complex. We adopted the methodology used to analyze BRCA2 mediator activity [36] and modified it to analyze yRad52 (Fig 1A). First, yRad52 was added to the preincubated yRPA-ΦX174 ssDNA complex and then hRAD51 was added to start formation of hRAD51-ssDNA complex. If yRad52 has mediator activity for hRAD51, it should facilitate formation of the hRAD51-ssDNA complex. To observe formation of the hRAD51-ssDNA complex, dsDNA was added exactly 5 min after the addition of the yRad51 to start the DNA strand exchange reaction. Unexpectedly, DNA strand exchange was stimulated by increasing amounts of yRad52 (Fig 1D and 1F). This result suggests that yRad52 works as a recombination mediator with hRAD51 *in vitro*.

**Fig 2:**
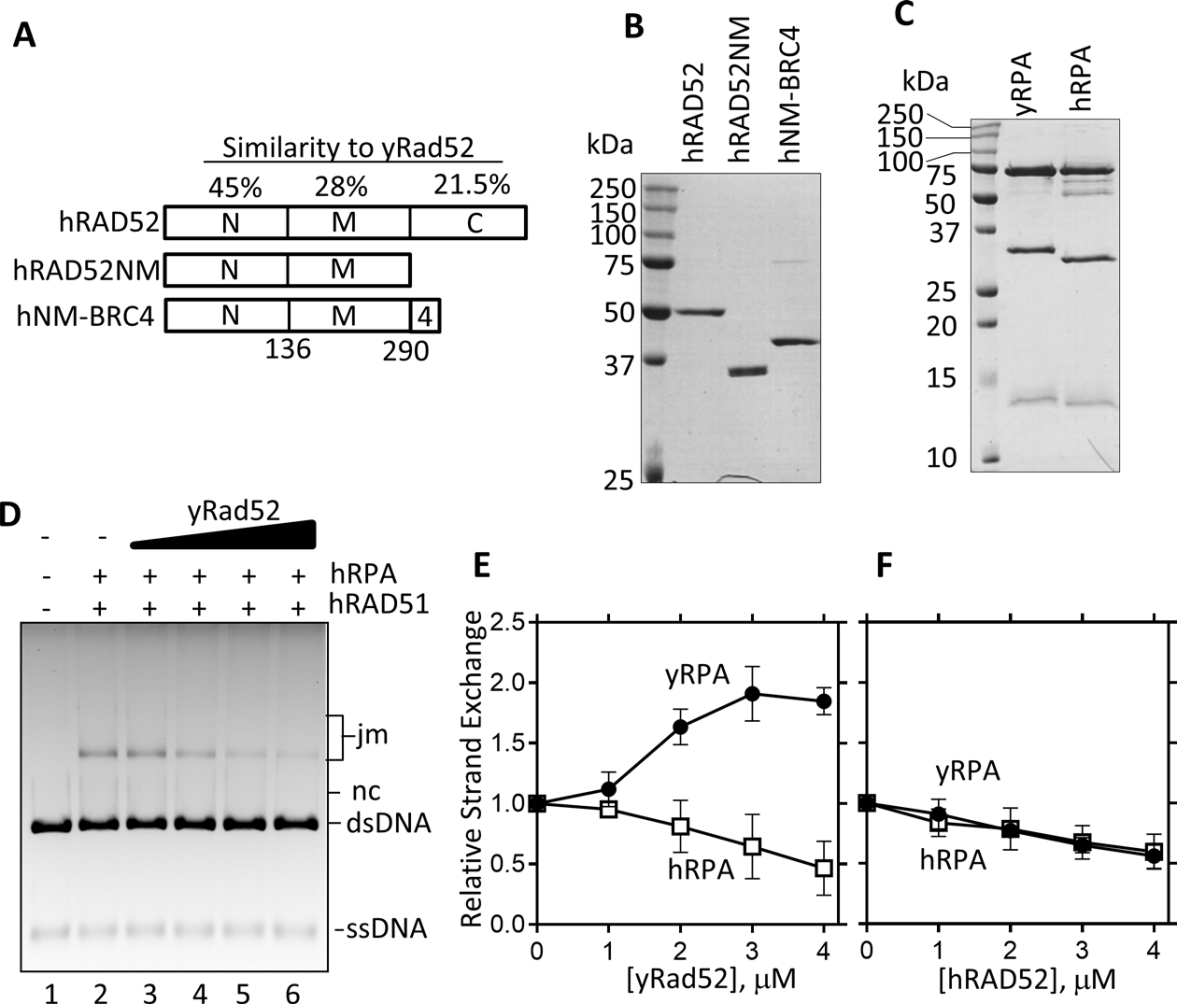
hRPA and hRAD52 failed to stimulate DNA strand exchange by hRAD51. (A) Regions of hRAD52 that correspond to yRad52N, M and C, and their similarities (%) to yRad52. (B and C) Same amount (2 μg) of purified hRAD52 and its derivatives (B), and yRPA and hRPA (C) were separated by SDS-PAGE and stained with coomassie brilliant blue. (D) DNA strand exchange assay using yRad52 and hRAD51 was performed as shown in Fig 1D, except that yRPA was replaced with hRPA. (E) Products of D were quantified (hRPA) and compared with the data shown in Fig 1F (yRPA). Error bars are standard deviations (n = 3). F. Same analyses as in E except that hRAD52 was used as a mediator.

The C-terminal yRad51 binding region of yRad52 is required for mediator activity [19, 26]. To investigate whether the same region is involved in the stimulation of DNA strand exchange by hRAD51, we constructed a derivative of yRad52 that lacked the C-terminal region (yRad52NM; Fig 1B and 1C). This derivative has been reported to lose the ability to interact yRad51 but retain binding activity to DNA and yRPA [18, 19, 37]. When yRad52NM was used, no stimulation of DNA strand exchange was observed (Fig 1E and 1F, “yRad52NM”), indicating that the C-terminal yRad51-binding region is required for this apparent mediator activity. It has been reported that yRad52 cannot form functional interaction with hRPA, and that this species-specific yRad52-yRPA interaction is required for mediator activity [24, 32]. To investigate whether the same interaction is involved in the stimulation of DNA strand exchange by hRAD51, we replaced yRPA with hRPA (Fig 2). yRad52 failed to recruit hRAD51 when ssDNA was complexed with hRPA (Fig 2D and 2E “hRPA”). Furthermore, consistent with the previously reported inability of hRAD52 to stimulate DNA strand exchange by hRAD51 [4, 27], hRAD52 did not stimulate the DNA strand exchange by hRAD51 with either hRPA or yRPA (Fig 2F). Based on these results, we concluded that yRad52 has an ability to recruit hRAD51 to the yRPA-ssDNA complex *in vitro* via an equivalent mechanism to the mediator. Consistently, we observed direct interaction between hRAD51 and yRad52 *in vitro* (Fig 1G).

### Effect of Rad52-BRC fusions on the mediator activity

**Fig 3:**
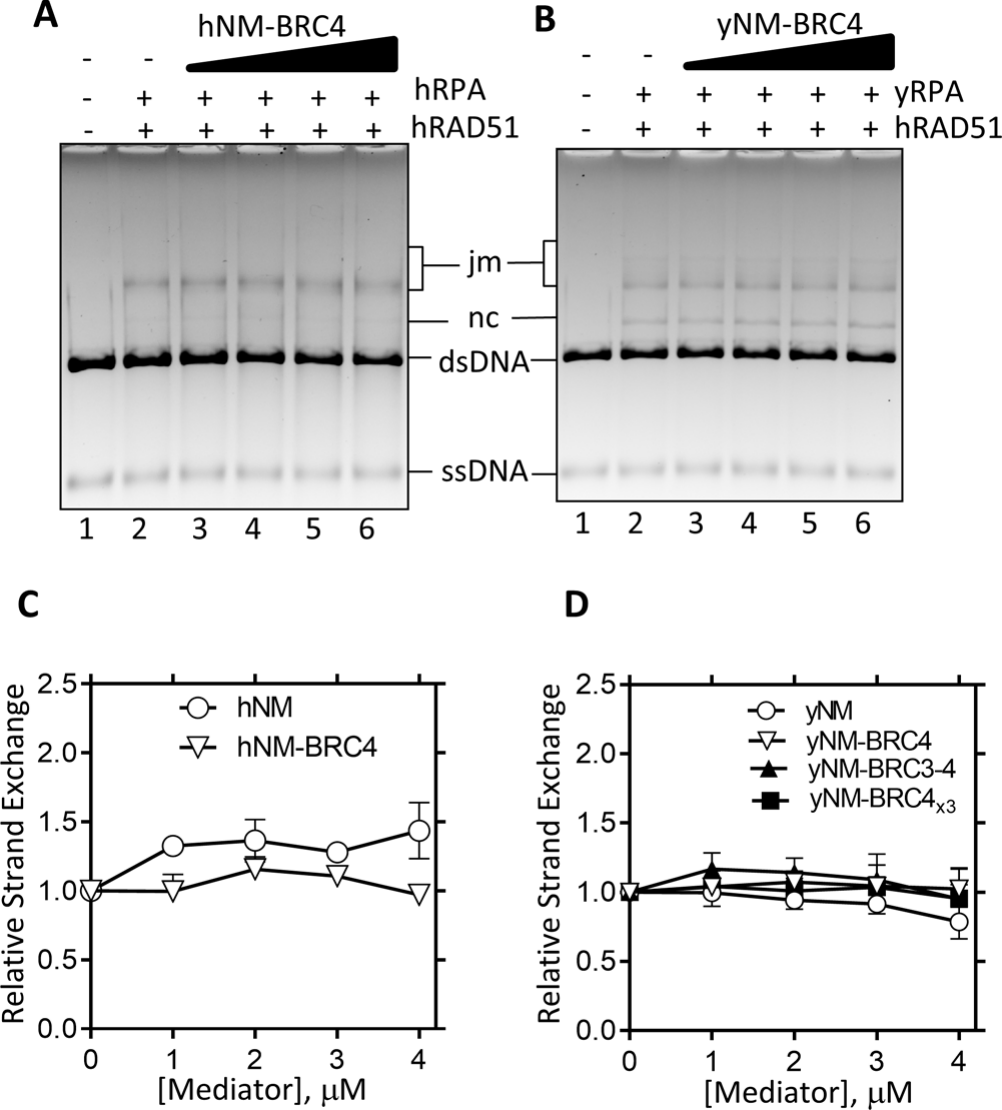
BRC repeats failed to replace the function of the C-terminal Rad51-binding domain of Rad52. (A and B) The mediator activity of hNM-BRC4 (A) and yNM-BRC4 (B) were examined in the presence of hRPA and yRPA, respectively. (C and D). The relative product formation of DNA strand exchange was plotted against the concentrations of the Rad52 derivatives. Error bars are standard deviations (n = 3).

Failure to detect the mediator activity of hRAD52 might be due to the lack of the functional interaction between the C-terminal region of hRAD52 and hRAD51. Therefore, we next tried to replace the C-terminal region of hRAD52 with a hRAD51-binding domain of BRCA2, a well-known human mediator. It has been demonstrated that BRCA2 has distinct hRAD51-binding regions called BRC repeats [31]. Because a single synthetic BRC repeat (BRC4; 35 amino acids) can interact with hRAD51 [38, 39], we constructed hRAD52NM-BRC4 fusion protein (Fig 2A, hNM-BRC4), and examined its mediator activity (Fig 3A and 3C). The hRAD52NM-BRC4 fusion protein did not stimulate DNA strand exchange by hRAD51 above the negative control (hRAD52NM).

We also constructed yRad52NM-BRC4 fusion protein, (yNM-BRC4; Fig 1B and 1C). Although intact yRad52 stimulated DNA strand exchange by hRAD51 (Fig 1D and 1F), replacement of its C-terminal Rad51-binding region with BRC4 eliminated the stimulation (Fig 3B and 3D, “yNM-BRC4”). We further tested yRad52NM fused with BRC3-BRC4 repeats (yNM-BRC3-4) and with three repeats of BRC4 (yNM-BRC4_x3_). Although it was reported that the multi-copy BRC repeats further enhanced the mediator activity of BRCA2 [40], none of these fusion proteins stimulated DNA strand exchange by hRAD51 (Fig 3D). Thus we concluded that BRC repeats cannot replace the Rad51-binding domain of Rad52 for mediator activity.

### The C-terminal region of yRad52, but not of hRAD52, is required for efficient ssDNA annealing

**Fig 4:**
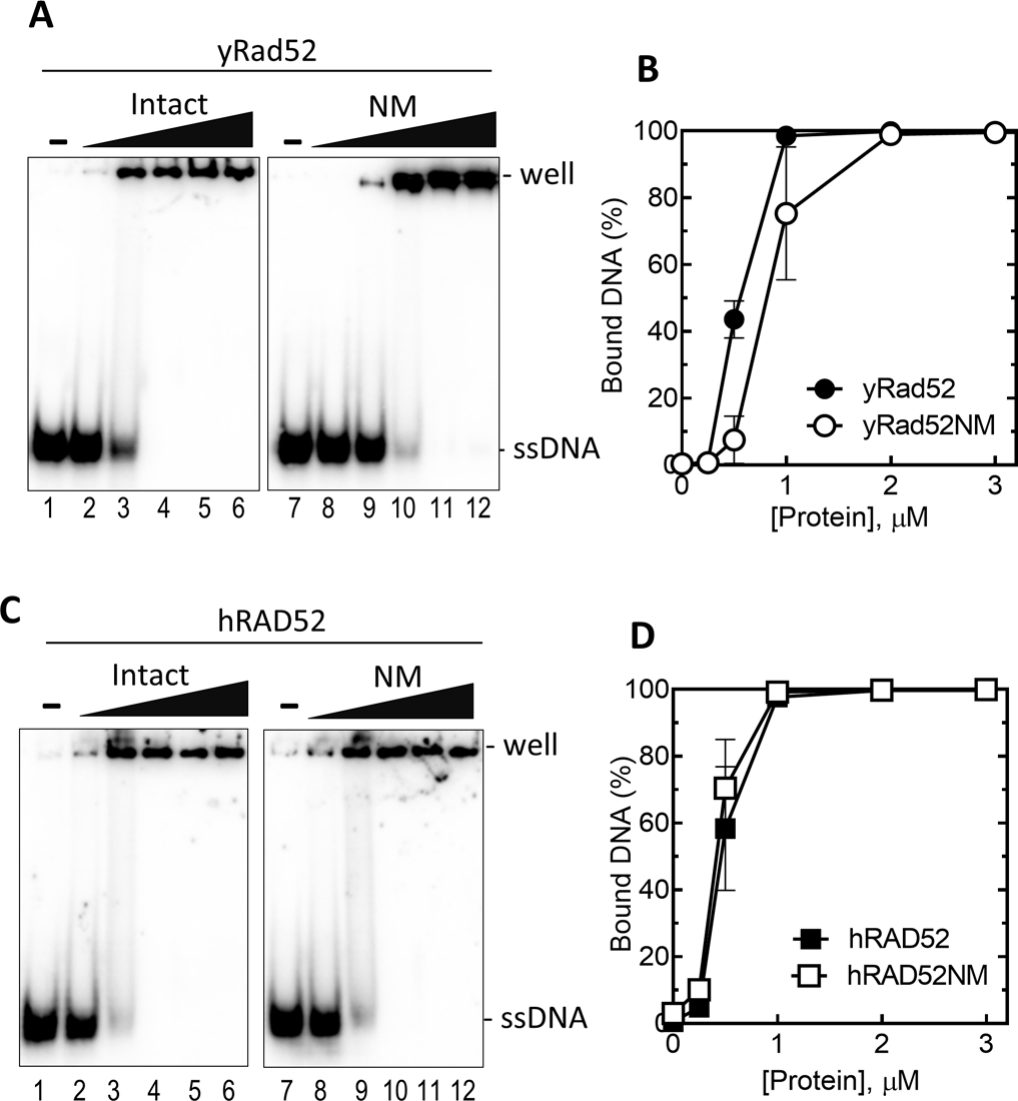
Effects of C-terminal deletion of yRad52 and hRAD52 on their ssDNA binding activities. (A and C) ^32^P-labeled 70-mer ssDNA (100 nM) was incubated without (lane 1 and 7) or increasing concentrations (0.25, 0.5, 1, 2 and 3 μM from left to right) of yRad52 (lane 2–6 of A), yRad52NM (lane 8–12 of A), hRAD52 (lane 2–6 of C), or hRAD52NM (lane 8–12 of C). Protein-ssDNA complexes were analyzed by electrophoresis through 6% polyacrylamide gel. (B and D) The same experiment in A and C were repeated and percentage of the ssDNA-protein complex was calculated from their band intensities. Error bases are STD (n = 3).

Seong et al.[18] reported that the C-terminal region of yRad52 harbors a “secondary” DNA binding site that is distinct from the “primary” site at the N-terminal region. A similar secondary DNA binding site was identified in hRAD52 [41] but mapped in a different region of the protein (amino acids 102–173). To compare the roles of the C-terminal region of yeast and human Rad52 in ssDNA binding, we carried out gel-mobility shift assay using Rad52 and Rad52NM from both yeast and human (Fig 4). When yRad52 and yRad52NM were incubated with ^32^P-labeled ssDNA, both proteins formed complexes with DNA (Fig 4A). However, quantification of the complexes showed a small but detectable difference in binding (Fig 4B). Approximately 1.5 to 2-fold more yRad52NM was required to saturate the ssDNA than yRad52, indicating that the C-terminal region is involved in the yRad52-ssDNA binding. On the other hand, the titration curves by hRAD52-ssDNA and hRAD52NM-ssDNA complex formations showed no detectable differences (Fig 4C and 4D), indicating that the C-terminal region of hRAD52 did not have prominent role in the ssDNA binding.

**Fig 5:**
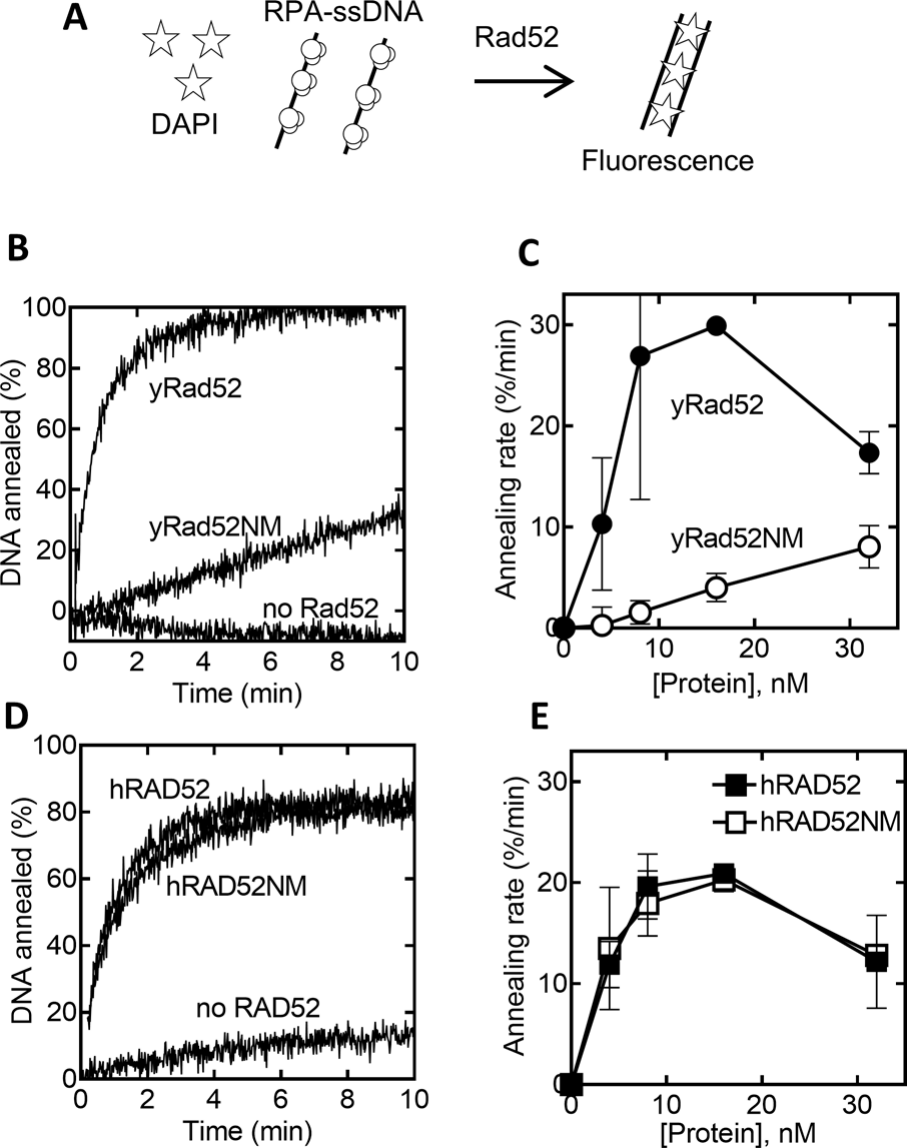
The C-terminal domain of hRAD52 but not of yRad52 is dispensable for ssDNA annealing. (A) Illustration of ssDNA annealing assay. The reaction contained DAPI, fluorescence of which increased upon binding to the dsDNA product. (B and D) Sixteen nanomolar of yRad52, yRad52NM, hRAD52, or hRAD52NM was added to complementary 70-nt ssDNA (2.86 nM each) that were pre-complexed with their cognate RPA (36 nM). (C and E). The same reactions as in B and D were repeated with various concentrations of the Rad52 derivatives and the initial rates of the reactions were plotted with the concentrations of Rad52 derivatives. Error bases are STD (n = 3 to 4).

Both yRad52 and hRAD52 anneal complementary ssDNA molecules [16, 24, 42, 43]. Previous studies have shown that the C-terminal region of Rad52 is not essential for annealing activity [19, 43]. To understand detailed contribution of the C-terminal regions of yRad52 and hRAD52 to annealing, we analyzed the ssDNA annealing activities of yRad52, hRAD52, and their C-terminal deletion derivatives (Fig 5). For quantitative kinetic analysis, annealing reactions were carried out in the presence of DAPI, which increase in fluorescence upon binding to dsDNA (Fig 5A). When yRad52 was added to complementary ssDNA molecules that were saturated with yRPA, DAPI fluorescence increased quickly, showing the efficient ssDNA annealing by yRad52 (Fig 5B “yRad52”). yRad52NM also mediated annealing, but the rate was much slower than the reaction carried out by yRad52 (Fig 5B and 5C). This result indicates that the C-terminal secondary DNA binding region is required for efficient annealing by yRad52. In contrast, hRAD52 and hRAD52NM showed indistinguishable annealing activities (Fig 5D and 5E), indicating that the corresponding region of hRAD52 is dispensable for ssDNA annealing.

## Discussion

Results presented in this paper (Figs 1 and 2) indicate that yRad52 can recruit hRAD51 onto yRPA-ssDNA complex *in vitro* by a mechanism equivalent to the mediator activity. This is surprising since humans have a Rad52 homolog that apparently does not have mediator activity. We speculate that, during evolution, the mediator activity of Rad52 was replaced by BRCA2 that recruits Rad51 in a distinct mechanism. Since BRCA2 is larger than Rad52 and interacts with many proteins, like BRCA1, PALB2 [44], and DSS1 [45], it can regulate HR more tightly than Rad52 does in yeast.

It was also unexpected that BRC repeats cannot replace the C-terminal region of yRad52 to re-constitute functional interaction with hRAD51 (Fig 3). Although our results do not exclude the possibility that BRC repeats failed to interact with hRAD51 when it is fused with our Rad52 constructs, we suggest that the binding of Rad51 and Rad52 is distinct form that of Rad51 and BRCA2. Crystallography has indicated that BRC4 associates with the Rad51 polymerization interface [46]. This region is not highly conserved in yeast and humans, but the corresponding region of yRad51 (N-terminal 150 amino acids) was mapped as the yRad52 interacting site by yeast two-hybrid [47]. These results suggest that both BRC4 and Rad52 might interact with the same or close regions of Rad51. More detailed structural information about Rad51-Rad52 interaction is needed to understand the exact role of the Rad52 C-terminus in mediator activity.

**Fig 6:**
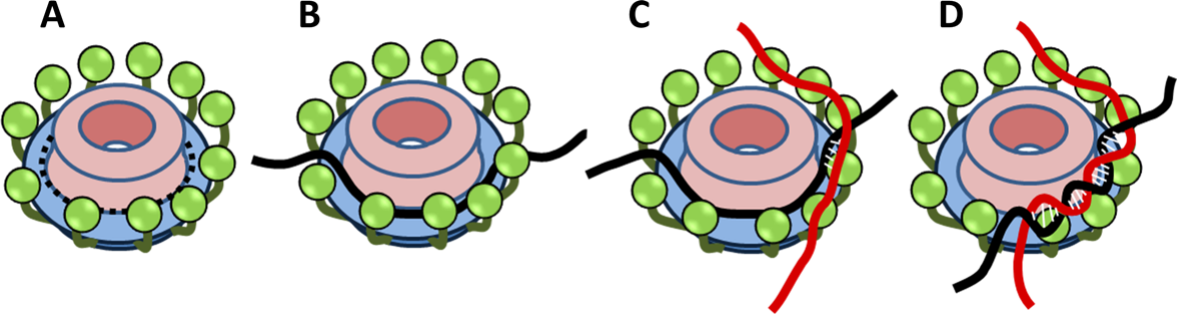
Model of ssDNA annealing by Rad52. Green spheres are hypothetical C-terminal regions of yRad52. (A) yRad52 ring structure that is expected from structural and biochemical evidence. (B to D) Proposed ssDNA annealing process by yRad52. See text for explanations.

Despite loss of mediator activity, ssDNA annealing activity is conserved in hRAD52 and yRad52. Our results indicate that yRad52 but not hRAD52 needs its C-terminal region for efficient ssDNA annealing (Fig 5). In addition, the C-terminal region is also involved in ssDNA binding of yRad52 but not of hRAD52 (Fig 4). It seems likely that the C-terminal region of yRad52 facilitates ssDNA annealing by interacting with the second DNA molecule. Based on this and other structural and biochemical evidence, we propose a model of yRad52-mediated annealing (Fig 6). Crystallography has revealed that N-terminal half of hRAD52 adopts an undecameric ring structure in which the ssDNA binding groove runs around the outside rim of the ring (dashed line in Fig 6A) [22, 26, 43]. Although the C-terminal region is not included in the crystal structure, we speculate that the C-terminal secondary DNA binding sites of yRad52 (green spheres in Fig 6) face to the primary ssDNA binding sites from outside of the ring. Electron micrographs of intact Rad52 showed ring-with-flaps structures that are consistent to our speculation [42, 48]. This arrangement can align two ssDNA molecules to facilitate annealing by yRad52 (Fig 6C and 6D). On the other hand, the C-terminal region of hRAD52 is not required for either ssDNA binding or annealing (Figs 4 and 5). This is consistent to the previous finding that the secondary DNA binding site of hRAD52 is not in the C-terminal but in the mid region of the protein and that the loss of the second DNA binding activity eliminated the activity of D-loop formation of hRAD52 [41]. Taken together, it is likely that both yRad52 and hRAD52 require two DNA binding sites for their maximum annealing activity, but that the secondary DNA binding sites are localized in different regions on their primary sequences.

## Supporting Information

S1 Fig Homologous alignment of yeast and human Rad52. The regions corresponding to the Rad52NM are enclosed in a box.
(PDF)

S1 Table Synthetic oligonucleotides used for this study. (PDF)
