# Supplementary material for: Roles of C-Terminal Region of Yeast and Human Rad52 in Rad51-Nucleoprotein Filament Formation and ssDNA Annealing
Source: PLoS One. 2016 Jun 30;11(6):e0158436. doi: 10.1371/journal.pone.0158436 (PMC4928909; doi:10.1371/journal.pone.0158436)
Supplement: S1 Fig — The regions corresponding to the Rad52NM are enclosed in a box. (PDF) [file pone.0158436.s001.pdf]

```
yRad52   1 ----MNEIMDMDEKKPVFGNHS--------------EDIQTKLDKKLGPE    32
               ...|:...:..|..|..|              :.||..|.::||||
hRad52   1 MSGTEEAILGGRDSHPAAGGGSVLCFGQCQYTAEEYQAIQKALRQRLGPE    50

yRad52  33 YISKRVGFGTSRIAYIEGWRVINLANQIFGYNGWSTEVKSVVIDFLDERQ    82
           |||.|:..|..::.||||.|||||||::||||||:..:....:||:|...
hRad52  51 YISSRMAGGGQKVCYIEGHRVINLANEMFGYNGWAHSITQQNVDFVDLNN   100

yRad52  83 GKFSIGCTAIVRVTLTSGTYREDIGYGTVENERRKPAAFERAKKSAVTDA   132
           |||.:|..|.|||.|..|:|.||:|||..|..:.|..:.|:|:|.||||.
hRad52 101 GKFYVGVCAFVRVQLKDGSYHEDVGYGVSEGLKSKALSLEKARKEAVTDG   150

yRad52 133 LKRSLRGFGNALGNCLYDKDFLAKIDKVKFD-PPDFDENNLFRPTDEIS-   180
           |||:||.||||||||:.|||:|..::|:... |.:.|.....|...|.|
hRad52 151 LKRALRSFGNALGNCILDKDYLRSLNKLPRQLPLEVDLTKAKRQDLEPSV   200

yRad52 181 ESSRTNTLHENQEQQQYPNKRRQLTKVTNTNPDSTKNLVKIENTVSRGTP   230
           |.:|.|:...|. ...:|    ||.:||:.:..|...:...::..||...
hRad52 201 EEARYNSCRPNM-ALGHP----QLQQVTSPSRPSHAVIPADQDCSSRSLS   245

yRad52 231 MMAAPAEANSKNSSNKDTDLKSLDASKQDQDDLLDDSLMFSDDFQDDDLI   280
           ..|..:||..:..      |:.....:|.::.:....:..|..
hRad52 246 SSAVESEATHQRK------LRQKQLQQQFRERMEKQQVRVSTP-------   282

yRad52 281 NMGNTNSNVLTTEKD------PVVAKQSPTASSNP--EAEQITFVTAK--   320
                     :.||.      |.|...:|...|.|  |.:.:..||.:
hRad52 283 ----------SAEKSEAAPPAPPVTHSTPVTVSEPLLEKDFLAGVTQELI   322

yRad52 321 ----------AATSVQNERYIGEESIFDPKYQAQSIRHTVDQTTSKHIPA   360
                     |.|....:..:...|..||...:.::.......|....|.
hRad52 323 KTLEDNSEKWAVTPDAGDGVVKPSSRADPAQTSDTLALNNQMVTQNRTPH   372

yRad52 361 SVLKDKTM----------------TT--------ARDSVYEKFAPKGKQL   386
           ||...|..                ||        ::|....|:.|.
hRad52 373 SVCHQKPQAKSGSWDLQTYSADQRTTGNWESHRKSQDMKKRKYDPS----   418

yRad52 387 SMKNNDKELGPHMLEGAGNQVPRETTPIKTNATAFPPAAAPRFAPPSKVV   436

hRad52 419 --------------------------------------------------   418

yRad52 437 HPNGNGAVPAVPQQRSTRREVGRPKINPLHARKPT    471
```

S1 Fig. Homologous alignment of yeast and human Rad52.
