# Supplementary material for: Roles of C-Terminal Region of Yeast and Human Rad52 in Rad51-Nucleoprotein Filament Formation and ssDNA Annealing
Source: PLoS One. 2016 Jun 30;11(6):e0158436. doi: 10.1371/journal.pone.0158436 (PMC4928909; doi:10.1371/journal.pone.0158436)
Supplement: S1 Table — (PDF) [file pone.0158436.s002.pdf]

S1 Table. Homologous alignment of yeast and human Rad52.

Name	Sequence
Pri-1	CCCAGATCTCAAAGAACCGACCCTGCTGGGCTTTCATACCGCGAGCG<br>GC
Pri-2	CCCGGATCCCTGTTCTTTTTCATCAAACAGGTTTTTCACTTTATCCAG<br>GCTTTCTTTCGCAATTTTCACTTTTTTGCCGCTCGCGGT
Pri-3	CCCCATATGATGAATGAAATTATGGATATGGATGAG
Pri-4	CCCCTCGAGCTGTTCTTTTTCATCAAACAGGT
Pri-5	CCCCATATGATGAATGAAATTATGGATATGGATGAG
Pri-6	TTTGGATCCTTCTCTGTCGTTAAAACATTACTG
Pri-7	CACCCGCATATGATGAATGAAATTATGGATATG
Pri-8	AAACTCGAGTCAGCTAATTAAGCTTAGTGATG
Pri-9	AAACATATGATGTCTGGGACTGAGGAAGCAATTCT
Pri-10	TTTCTCGAGAGATGGATCATATTTCCTTTTCTTCATG
Pri-11	TTTCTCGAGGGATCCCGCTGCCTCACTCTTCTC
BRC3<br>(hybridized)	GATCCCTTTGAAACCAGCGATACCTTTTTTCAGACCGCGAGCGGCAA<br>AAACATTAGCGTGGCGAAAGAAAGCTTTAACAAAATTGTGAACTTTTT<br>TGATCAGAAACCGGAACA
	TATGTTCCGGTTTCTGATCAAAAAAGTTCACAATTTTGTTAAAGCTTTC<br>TTTCGCCACGCTAATGTTTTTGCCGCTCGCGGTCTGAAAAAAGGTATC<br>GCTGGTTTCAAAGG
BRC4a<br>(hybridized)	TATGAAAGAACCGACCCTGCTGGGCTTTCATACCGCGAGCGGCAAAA<br>AAGTGAAAATTGCGAAAGAAAGCCTGGATAAAGTGAAAAACCTGTTTG<br>ATGAAAAAGAACAGC
	TCGAGCTGTTCTTTTTCATCAAACAGGTTTTTCACTTTATCCAGGCTTT<br>CTTTCGCAATTTTCACTTTTTTGCCGCTCGCGGTATGAAAGCCCAGCA<br>GGGTCGGTTCTTTCA
BRC4b<br>(hybridized)	GATCTAAAGAACCGACCCTGCTGGGCTTTCATACCGCGAGCGGCAAA<br>AAAGTGAAAATTGCGAAAGAAAGCCTGGATAAAGTGAAAAACCTGTTT<br>GATGAAAAAGAACAGG
	GATCCCTGTTCTTTTTCATCAAACAGGTTTTTCACTTTATCCAGGCTTT<br>CTTTCGCAATTTTCACTTTTTTGCCGCTCGCGGTATGAAAGCCCAGCA<br>GGGTCGGTTCTTTA
TSO252	TGGTTGAGTACTCACCAGTCACAGAAAAGCATCTTACGGATGGCATG<br>ACAGTAAGAGAATTATGCAGTGC
TSO253	GCACTGCATAATTCTCTTACTGTCATGCCATCCGTAAGATGCTTTTCT<br>GTGACTGGTGAGTACTCAACCA
